# Kleine-Levin Syndrome in an 8-Year-Old Girl with Autistic Disorder: Does Autism Account a Primary or Secondary Cause?

**Published:** 2015

**Authors:** Mitra HAKIM SHOUSHTARI, Mirfarhad GHALEBANDI, Azita TAVASOLI, Maryam POURSHAMS

**Affiliations:** 1 Mental Health Research Center, Iran University of Medical Sciences, Tehran, Iran; 2 Department of Pediatric Neurology, Ali-Asghar Children’s Hospital, Tehran, Iran

**Keywords:** Kleine-Levin syndrome, Autistic spectrum disorder, Epilepsy

## Abstract

**Objective**

Kleine–Levin syndrome (KLS) is a rare disorder with an unknown etiology. Autism spectrum disorder is characterized by various degrees of impairment in social communication, repetitive behavior and restricted interests. Only four patients of KLS with autistic spectrum disorder (ASD) have been reported so far. This report presents an 8-year-old girl with history of autistic disorder and epilepsy that superimposed KLS. Because of the rarity of KLS and related studies did not address whether autism accounts for a primary or secondary cause, the area required attention further studies.

## Introduction

Kleine–Levin syndrome (KLS) is a rare disorder characterized by relapsingremitting episodes of various degrees of hypersomnia, behavioral disturbance, cognitive impairment, binge eating (rapid consumption of a large amount of food) and hypersexuality (1). One meta-analysis reported that the median age at onset of the disease was 15 years (range, 4–80 yr). However, the onset the disease in 81% of the patients was during the beginning of the second decade of their ages (2). Each episode takes about one or two weeks and generally the patients do not experience any symptoms between episodes. The exact cause is not well identified but hypothalamic dysfunction has been proposed. Relapses occur every few weeks or months, the condition may last for a decade or more and recovery usually occurs spontaneously (3). Between episodes, sleep patterns, cognition, mood, and eating habits are usually normal. When episodes occur, electroencephalography might show diffused or local slowing of activity. Functional imaging studies have revealed hypoactivity in thalamic, hypothalamic and frontotempoal regions (4). Approximately 60% of the cases have precipitating factors such as infections, head trauma and alcohol consumption. Common symptoms were hypersomnia, cognitive changes, derealization, eating disturbances, hypersexuality, compulsive behaviors, and depressed mood (5). In a systematic review, stimulants (only amphetamines) were effective in decreasing somnolence in 40% of cases. Among the mood stabilizers, only lithium had effectiveness for controlling relapses when compared to medical abstention. Electroconvulsive therapy, insulin coma therapy, narcoleptics and antidepressants have not been beneficial. Patients with secondary KLS were old in age and had more severe form of disease while they had similar symptoms and response to treatment compared to patients with primary KLS (6). Furthermore, another literature review carried out previously by the authors of this study have found only four Kleine-Levin syndrome patients with autistic spectrum disorder (ASD). That is, two of them were adolescents with Asperger syndrome diagnosed in 1992 and the remaining two were with autistic disorder diagnosed in 2009 (7, 8). This report presents an 8-year-old female with a history of autistic disorder and epilepsy superimposed by KLS.

## Case Report

An eight-year- old girl was referred to child psychiatrist four years ago because of her problems in talking. She had echolalia and talked out of context. Her mother reported that she was a full-term baby delivered by caesarean section with no complication. She lived at home with her parents and one younger brother. When she was 2 months old, she started to show first seizure episodes. Electroencephalography (EEG) showed evidence of epileptic form activity. Child neurologist could not find any underlying cause for her seizure disorder. History of perinatal hypoxic ischemic encephalopathy (HIE) and seizure disorder in her family were negative. The results of laboratory tests including complete blood count, biochemistry and metabolic tests were normal. Physical examination of the infant did also not show any cutaneous lesion in favor of neurocutaneous disease or other signs of underlying disorders. Written informed consent was obtained from the patient’s parents for the publication of this report. Despite treatment with phenobarbital, seizure episodes were continued and by the age of 8 months, her neurologic status worsened with excessive irritability and jitteriness. She had recurrent episodes of body tightening with arms and legs pulled in tightly accompanied by loud grunts, eye blinking, gagging and jerking movements. Valproate was added to phenobarbital that led to some decrease in seizure frequency. However, by the age of 14 months, when she started to walk, she had developed truncal ataxia and tremors. On evaluating her developmental milestones, her motor development was within normal limits. She began to vocalize at the age of 3 months but could not develop any words until the age of 3 years. She began to show some motor stereotypies, including movement in hands and swinging at the age of 3 years. She was hyperactive and did not pay attention to risks. She had some inappropriate fears such as fear of wind and birds and smelled everything. She had some difficulties in social interaction such as poor eye contact and failure to engage in imitative games. She could not be able to paint and did not recognize colors. She had no interest to play with other children but liked to look at a washing machine while it was working, jumping and screaming too much. She was not socially responsive, did not smile responsively and repeated something with herself. She did not show sensitivity to temperature, and had two scars on her arms due to burn with boiled water. She was diagnosed to have autistic disorder based on DSM-IV criteria. To lower her stereotypies and hyperactivity, she was started with risperidone and the dose was increased to 3 mg/day. On the next follow up session although the previous symptoms were decreased but she became very aggressive towards children and she was prescribed to take clonidine 0.1 mg per day. She started to show some improvements in social and communication skills. Then, occupational and speech therapy were added to help improve her skills. By the end of the year, she has developed recurrent episodic movement in her neck and eyes. Suspecting that she had seizures, she was given oxcarbazepine 300 mg daily. Hence, the abnormal movements were controlled after 3 weeks treatment. In all these conditions, the child has been continued treatment with valproate and phenobarbital. By the age of one year, she developed recurrent episodes of excessive sleep associated with an abrupt onset of hyperphagia, increased irritability, masturbation, self-laughing and talkativeness lasting for 2 days. She experienced three episodes with complete recovery between them. There was no positive family history of neurological or psychiatric disorder. All biochemical, endocrine and imaging parameters were found within normal range. Electroencephalography (EEG) was like previous one and did not show any changes. A clinical diagnosis of KLS was made, and patient was put on lithium 300 mg twice a day. She has been adherent this treatment and became symptom free since the past 6 months.

## Discussion

Autism spectrum disorder is characterized by various degrees of impairment in social communication, repetitive behavior and restricted interests (9). The prevalence of epilepsy in autism ranges from 7% to 46% (10) and prevalence of sleep problems in children with autism spectrum disorder is estimated to be 40 to 80% of the affected children (11). Such children commonly have disturbed and irregular sleep patterns –wake patterns, early morning awakening, and behavioral problem at bedtime consisting of unusual bedtime routines (12). A study conducted in 2006 reported that approximately 86% of children with ASD had at least one sleep problem including bedtime resistance, insomnia, parasomnias, sleep disordered breathing, morning rise problems and daytime sleepiness. Young age, hypersensitivity to stimulus, co-sleeping, epilepsy, comorbid attention deficit hyperactivity disorder (ADHD), asthma, allergy, gastrointestinal symptoms, bedtime ritual, use of medication, and family history of sleep problems were related to sleep problems. Comorbid disorders including epilepsy, insomnia, and parasomnias increase the risk of daytime sleepiness (13). The etiology of KLS is unknown. The findings from a cohort study on 108 subjects with KLS reported that 5% of patients had family history of KLS, 25% had a complicated birth history and another 15% had some degree of developmental delay (14). In addition, a study revealed only18 subject to have comorbidities out of 186 patients with KLS (6). Klein-Levin syndrome is now believed to be an autoimmune disorder. The association of KLS with histocompatibility antigen DQBI 0201 and the occasional precipitation of the disorder after systemic infection as well as the relapsing and remitting nature of the disorder may suggest an autoimmune etiology (14). An epileptic etiology by EEG studies and the lack of efficiency in the antiepileptic drugs during episodes have been identified (15). Our study demonstrated that the symptoms of KLS have appeared in the presence of taking phenobarnital, valproate and oxcarbazepine, Therefore, epileptic activity was not the reason. The occurrence of KLS with preexisting ASD raised a question whether autism accounts for a primary or secondary cause of KLA. The rare in occurrence of the disorder especially in females and during the young age invited the attention of the researchers. If the child’s underlying disorder were considered as accounting for secondary KLS, it would be more unusual because such cases are expected to happen in older age than as in primary KLS. Because KLS is a rare disorder and related studies did not address the gap, the area requires further studies. Stimulants have been proposed for the treatment of KLS (6). However, we did not use them in treating the child because they can lower the seizure threshold (9). Besides, although the symptoms of KLS were controlled by lithium, long-term follow-up therapies have not been described because the condition is rare (16).

## Notes

**Authors’ contributions**: MHSH and AT managed the case. MHSH and MP drafted the paper and scientific editing of the manuscript. MGH read and approved the final manuscript.

## References

[1] Hauri P, American Academy of Sleep Medicine (2005). The International Classification of Sleep Disorders—Revised Diagnostic and Coding Manual.

[2] Huang YS, Arnulf I (2006). The Kleine-Levin Syndrome. Sleep Med Clin.

[3] Mudgal S, Jiloha RC, Kandpal M, Das (2009). Kleine-Levin syndrome. Pract Neurol.

[4] Arnulf I, Rico TJ, Mignot E (2012). Diagnosis, disease course, and management of patients with Kleine-Levin syndrome. Lancet Neurol.

[5] Sourav Das MDa, Ravi Gupta (2014). Kleine-Levin Syndrome: A Case Report and Review of Literature. Pediatric Neurology.

[6] Arnulf I, Zeitzer JM, File J, Farber N, Mignot E (2005). Kleine-Levin syndrome: a systematic review of 186 cases in the literature. Brain.

[7] MArcelo L, Berthier, Juan Santamaria, Horacio Encabo (1992). Recurrent Hypersomnia in Two Adolescent Males with Asperger’s Syndrome. J Am Academy Child Adoles Psychiatr.

[8] Nahit Motavalli Mukaddesi, Fateh R, Kilincaslan A (2009). KleineLevin syndrome in two subjects with diagnosis of autistic disorder. World J Biol Psychiatry.

[9] ( 2011). Kaplan and Sadock’s Synopsis of Psychiatry: Behavioral Sciences, Clinical Psychiatry.

[10] Masayuki Sasaki (2015). SPECT ﬁndings in autism spectrum disorders and medically refractory seizures. Epilepsy Behav.

[11] Souders MC, Mason TB, Valladares O (2009). Sleep behaviors and sleep quality in children with autism Spectrum disorders. Sleep.

[12] Richdale AL (2006). Sleep Problems in Children with Autism and in Typically Developing Children. Focus Autism Other Dev Disabl.

[13] Liu X, Hubbard JA, Fabes RA, Adam JB (2006). Sleep Disturbances and Correlates of Children with Autism Spectrum Disorders. Child Psychiatry Human Development.

[14] Kotagal S, Kenneth S, Stephen A, Donna F, Nina S (2012). Sleep-Wake Disorders. Swaiman’s Pediatric Neurology.

[15] Arnulf I, Rico TJ, Mignot E (2012). Diagnosis, disease course, and management of patients with Kleine-Levin syndrome. Lancet Neurol.

[16] Oliveira MM, Conti C, Prado GF Pharmacological treatment for Kleine-Levin syndrome (Review). Cochrane Database of Systematic Reviews (CDSR).

